# Leitlinientreue und Qualitätssicherung in der bildgebenden Diagnostik bei Verdacht auf Kindesmisshandlung in Deutschland

**DOI:** 10.1007/s00117-021-00872-w

**Published:** 2021-07-02

**Authors:** Susanne Dargel, Martin Stenzel, Brigitte Stöver, Ekkehard Schleußner, Daniel Wittschieber, Sibylle Banaschak, Hans-Joachim Mentzel

**Affiliations:** 1 Sektion Kinderradiologie, Institut für Diagnostische und Interventionelle Radiologie, Universitätsklinikum, Am Klinikum 1, 07747 Jena, Deutschland; 2 Abteilung für Kinderradiologie, Kliniken Köln gGmbH, Kinderkrankenhaus Amsterdamer Straße, Köln, Deutschland; 3 em. Abteilung Pädiatrische Radiologie, Charité Universitätsmedizin, Berlin, Deutschland; 4 Klinik für Geburtsmedizin, Department für Frauenheilkunde und Geburtsmedizin, Universitätsklinikum, Jena, Deutschland; 5 Institut für Rechtsmedizin, Universitätsklinikum, Jena, Deutschland; 6 Institut für Rechtsmedizin, Uniklinik Köln, Köln, Deutschland

**Keywords:** Kindesmisshandlung, Nicht akzidentielles Trauma, Röntgen, Skelettscreening, Qualitätssicherung, Child abuse, Non accidential trauma, Radiography, Skeletal survey, Quality control

## Abstract

**Hintergrund:**

Die bildgebende Diagnostik nimmt in der Evaluation nichtakzidentieller Verletzungsfolgen im Kindesalter eine Schlüsselrolle ein. Frakturen sind nach Hautläsionen wie Abschürfungen oder Hämatomen die zweithäufigste Folge körperlicher Kindesmisshandlung. Mit Hilfe radiologischer Kriterien können nichtakzidentielle von akzidentiellen Frakturen differenziert werden. Spezielle Frakturtypen wie die klassische metaphysäre Läsion können nur bei hoher Bildqualität differenziert werden.

**Fragestellung:**

In einer prospektiven Analyse sollten Leitlinientreue und Qualitätssicherung der radiologischen Diagnostik bei Misshandlungsverdacht in Deutschland erfasst werden. Dazu wurden Quantität und diagnostische Qualität in der universitären und nichtuniversitären Versorgung sowie in Abhängigkeit einer vorhandenen kinderradiologischen Fachabteilung analysiert.

**Material und Methoden:**

Es wurden 958 Röntgenuntersuchungen von 114 vermuteten Misshandlungsfällen (46 Mädchen, 68 Jungen) bewertet. Insgesamt 42 Fälle aus universitären, 42 aus maximalversorgenden und 30 aus regelversorgenden Kliniken mit einem medianen Alter von 6 Monaten (3 Wochen – 3. Lebensjahr) wurden als DICOM-Daten von 3 Kinderradiologen im Konsensverfahren hinsichtlich Leitlinientreue und verschiedener Qualitätsparameter beurteilt. Ein Begleitfragebogen sollte die theoretischen Kenntnisse mit der jeweiligen praktischen Umsetzung vergleichen.

**Ergebnisse:**

Je Fall wurden im Mittel 8,4 Röntgenaufnahmen (1–22) angefertigt. In 12 von 114 beurteilten Fällen (10 %) lag ein vollständiger Skelettstatus nach S1-Leitlinie GPR vor. In 13 Fällen (10,5 %) wurde ein Babygramm durchgeführt. Abteilungen mit kinderradiologischem Schwerpunkt fertigten signifikant mehr Röntgenaufnahmen je Skelettstatus an als Einrichtungen ohne Schwerpunkt (*p* < 0,05). Eine signifikant höhere qualitative Umsetzung wurde in Universitätskliniken verzeichnet (*p* < 0,001). Eine Übereinstimmung von Fragebogenantwort und vorliegendem Bildmaterial zeigte sich unabhängig der Institutionsart nur marginal.

**Diskussion:**

In Deutschland fehlt bislang mehrheitlich ein leitliniengerechtes Vorgehen bei Misshandlungsverdacht. Es bleibt abzuwarten, ob sich dies mit der breiteren Implementierung von Kinderschutzgruppen und der 2019 verabschiedeten S3-Kinderschutzleitlinie (AWMF-Registrierungsnummer: 027-069) zukünftig ändern wird. Die Etablierung von Referenzzentren für Zweitbefundung und Empfehlungen zur Aufnahmetechnik können zusätzlich die Versorgungsqualität nachhaltig verbessern.

## Abstract (en)

**Background:**

Diagnostic imaging plays a key role in the evaluation of non-accidental consequences of injuries in childhood. Fractures are the second most common consequence of child abuse, after skin lesions such as abrasions or bleeding. With the evidence of radiological criteria, non-accidental fractures can be differentiated from accidental fractures. Special types of fractures such as the classic metaphyseal lesion can only be differentiated if the image quality is high.

**Aim:**

The goal of this prospective study was to assess adherence to guidelines and quality assurance of radiological diagnostics in the event of suspected abuse in Germany. For this purpose, the quantity and diagnostic quality in university and non-university hospitals as well as the existence of a pediatric radiology department were analyzed.

**Materials and methods:**

In all, 958 X‑ray examinations of 114 suspected abuse cases (46 girls, 68 boys) were evaluated; 42 cases from university, 42 from maximum care and 30 from regular care clinics with a median age of 6 months (3 weeks—3 years of age) were assessed as DICOM data by 3 pediatric radiologists in a consensus procedure with regard to adherence to guidelines and various quality parameters. An accompanying questionnaire was used to compare the theoretical knowledge with the respective practical implementation.

**Results:**

A mean of 8.4 X‑rays (range 1–22) were made per case. In 12 of 114 assessed cases (10%) there was a complete skeletal status according to the S1 guideline. A babygram was performed in 13 cases (10.5%). Departments with focus on pediatric radiology produced significantly more X‑rays per skeletal status than facilities without this specialization (*p* < 0.04). Significantly higher qualitative implementation was recorded in university hospitals (*p* < 0.001). Regardless of the type of institution, there was only marginal agreement between the questionnaire response and the available image material.

**Conclusion:**

In Germany, a guideline-compliant procedure in the event of suspected child abuse is largely lacking. It remains to be seen whether this will change in the future with the broader implementation of child protection groups and the S3+ child protection guideline adopted in 2019 (AWMF register 027-069). The establishment of reference centers for a second diagnosis and recommendations for imaging technology can also improve the quality of care over the long term.

## Hintergrund und Fragestellung

In den Industrienationen sterben jährlich ca. 3500 Kinder an den Folgen körperlicher Misshandlung. Nach UNICEF(United Nations Children’s Fund)-Schätzungen ist pro Todesfall von ca. 150 weiteren Fällen körperlicher Gewalt auszugehen [1]. In Deutschland wird für 2019 eine Zahl von 4055 Fällen mit Kindesmisshandlung angegeben (§ 225 Strafgesetzbuch [StGB]), 112 Kinder wurden Opfer eines Tötungsdeliktes [2]; 90 % der misshandelnden Kinder sind jünger als 5 Jahre, 55 % jünger als 1 Jahr.

Generell wird zwischen akzidentellen (z. B. Verkehrsunfall) und nichtakzidentellen Verletzungen als Folge grober körperlicher Gewalt durch Betreuungspersonen unterschieden. Anhand typischer Befundkonstellationen und ggf. widersprüchlicher Angaben hinsichtlich des Unfallhergangs ist eine Kindesmisshandlung nachzuweisen oder auszuschließen. Neben körperlicher Untersuchung und Beurteilung des Augenhintergrundes nimmt die Röntgenuntersuchung des Skeletts eine wesentliche Rolle ein [3].

Das Röntgen-Skelettscreening ist in internationalen Leitlinien in unterschiedlichem Umfang obligatorisch festgelegt [3–7]. Es beinhaltet standardisiert durchzuführende Röntgenaufnahmen, die wesentliche Skelettelemente abbilden. Diese werden bei entsprechendem Verdacht durch gezielte Röntgenaufnahmen (in 2 Ebenen) ergänzt. Untersuchungsmethoden wie Skelettszintigraphie oder Ganzkörper-Magnetresonanztomographie (MRT) wird eine ergänzende Rolle in der Evaluation der nichtakzidentiellen Traumafolgen im Kindesalter zugewiesen. Trotz bestehender Evidenz der Skelettszintigraphie für nichtdislozierte und subtile Rippenfrakturen wird diese Untersuchung auf Grund der Strahlenbelastung nicht empfohlen, kann im individuellen Fall jedoch nach ausreichender Abwägung durchgeführt werden. Für die Ganzkörper-MRT fehlt ausreichende Evidenz. Die Sensitivität für missbrauchsspezifische Verletzungsmuster wie Rippenfrakturen ist gering, sodass ein routinemäßiger Einsatz dieser Technik nicht zu rechtfertigen ist [8, 9].

Trotz vorhandener Leitlinienempfehlungen weisen die in der täglichen Praxis angefertigten Röntgenaufnahmen häufig Mängel auf. Diese können zu erheblichen Folgen für das Wohl des Kindes führen [10–15]. Eine sorgfältige Bildgebung kann wegweisende und eindeutige Belege für eine nichtakzidentielle Verletzung und eventuelle Mehrzeitigkeit des Geschehens hervorbringen. Nur bei Durchführung nach aktuellem Stand der Wissenschaft kann die volle forensische Beweiskraft entfaltet und bei Gerichtsprozessen zur Determinierung des Strafmaßes genutzt werden [16].

**Figure 1:**
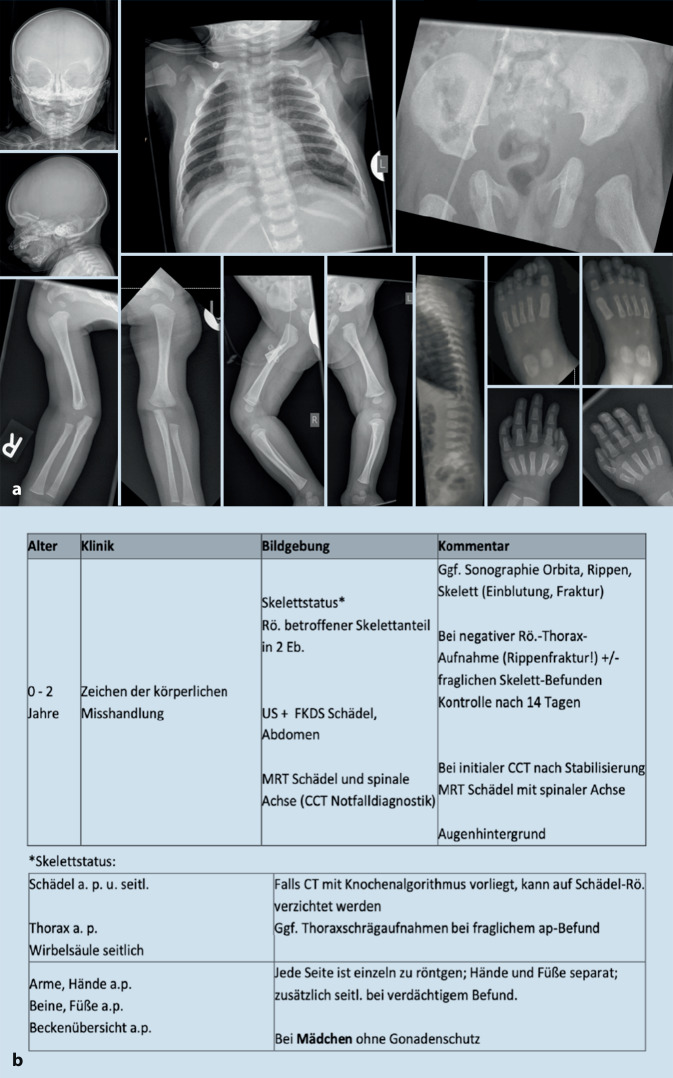

Ziel der Studie war es, die in der Bundesrepublik Deutschland bei Misshandlungsverdacht durchgeführte radiologische Diagnostik auf Einhalten der Leitlinie sowie Reliabilität zu untersuchen. Zum Zeitpunkt der Untersuchung galt die S1-Leitlinie der Gesellschaft für Pädiatrische Radiologie (GPR; AWMF 064-014), deren wesentliche Anforderungen in Abb. 1 dargestellt ist. Folgende Fragen sollten beantwortet werden:

- Wurde der vorliegende Skelettstatus nach Leitlinie durchgeführt?
- Sind quantitative und qualitative Unterschiede je nach Art der Institution – Regel‑, Maximalversorgung oder Universitäten – sowie in Abhängigkeit einer vorhandenen kinderradiologischen Fachabteilung zu verzeichnen?
- Zeigen sich Diskrepanzen zwischen den Angaben im Begleitfragebogen und der praktischen Umsetzung des Skelettstatus?

## Studiendesign und Untersuchungsmethoden

Die prospektiv konzipierte Studie umfasste einen Zeitraum von 22 Monaten – von 1. Oktober 2009 bis 31. Juli 2011. Insgesamt 1050 Mitglieder wissenschaftlicher Fachgesellschaften (Deutsche Gesellschaft für Kinder- und Jugendmedizin; Deutsche Gesellschaft für Kinderchirurgie; Gesellschaft für Pädiatrische Radiologie) wurden postalisch und per E‑Mail über Durchführung und Inhalt der Studie informiert und zur Teilnahme ermuntert. Ein Reminder zur laufenden Studie erfolgte 6 Monate vor Ende der Laufzeit. Als Einschlusskriterien wurde ein Alter bis zum vollendeten dritten Lebensjahr, das Vorliegen konventioneller Röntgendiagnostik mit klinischer Fragestellung „battered child“, Kindesmisshandlung oder nichtakzidentelles Trauma und die Dokumentation als DICOM-Daten festgelegt. Der auszufüllende Begleitfragebogen enthielt Daten zur Art der Institution sowie die Möglichkeit für Angaben, warum von der Leitlinie abgewichen wurde. Ein Votum der zuständigen Ethikkommission lag vor (2574-06/09), die Deklaration von Helsinki wurde beachtet.

Die pseudonymisierten Röntgenaufnahmen (Alter, Geschlecht bekannt) wurden durch zwei Untersucher mit langjähriger und einen Untersucher mit vierjähriger kinderradiologischer Expertise auf dem Gebiet der forensischen Radiologie im Konsensus ausgewertet. Fälle der eigenen Institution wurden im Sinne der Verblindung nur durch die anderen beiden Gutachter bewertet. Zur Bildanalyse wurden nach DIN-Norm (6868-13) Röntgenbefundungsmonitore (BARCO, Karlsruhe, Deutschland) eingesetzt. Vorliegende Röntgenaufnahmen wurden hinsichtlich ihres Abbildungsumfangs bewertet und in Bezug zur damals geltenden S1-Leitlinie der Gesellschaft für Pädiatrische Radiologie erfasst (AWMF 064-014).

**Figure 2:**
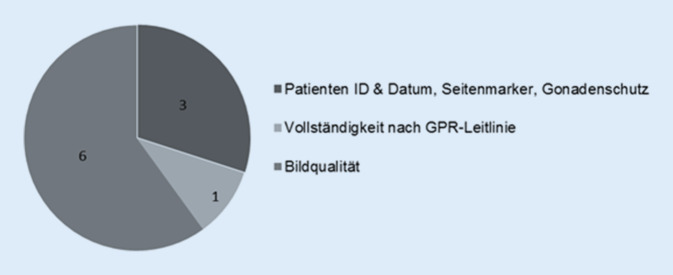

Um einen Vergleich in qualitativer Sicht zu ermöglichen, wurde ein Scoringsystem etabliert. Jeder abgebildete Skelettabschnitt wurde nach Teilaspekten der Bildqualität beurteilt (Abbildungsumfang, Verprojezierung, Fehlbelichtung, Bewegungsunschärfe, Dezentrierung, Bildrauschen, Überlagerung, kritische Strukturen, Einblendung). Die Bewertung der Qualitätsparameter (kritische Strukturen, Einblendung, Überlagerung u. a.) durch das Studienteam fand nach den Kriterien der Leitlinie zur Qualitätssicherung in der Röntgendiagnostik der Bundesärztekammer (BÄK-Leitlinie) statt [17]. Die qualitative Beurteilung ging mit maximal 6 Punkten in den Score ein. Vollständigkeit nach Leitlinie sowie die Beachtung allgemeiner Bildparameter wie Patienten-ID, Seitenmarker und die Verwendung eines Gonadenschutzes umfassten 4 Scorepunkte. Insgesamt konnten 10 Punkte erreicht werden (Abb. 2).

**Figure 3:**
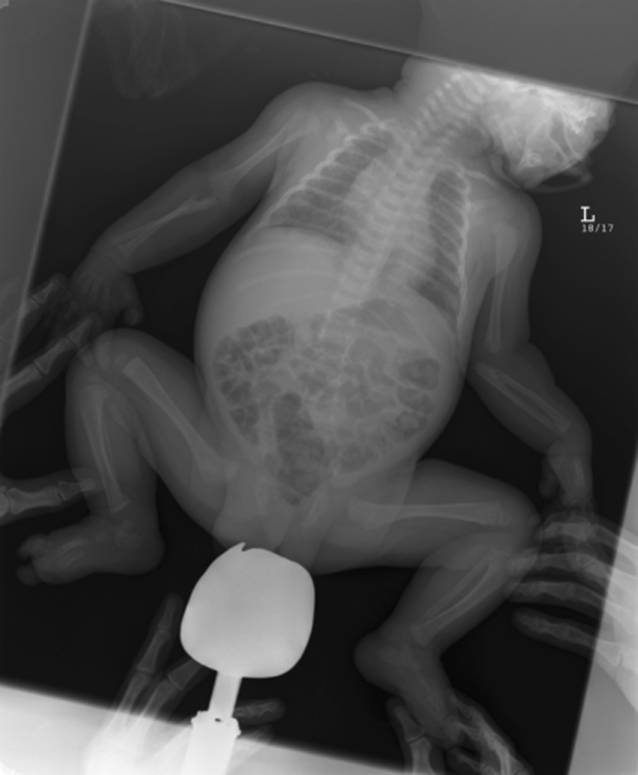

Lag der Skelettstatus lediglich in Form eines Babygrammes vor, wurde die Scorezahl halbiert. Als Babygramm werden die Röntgenaufnahmen gewertet, auf denen das gesamte Kind bzw. die obere oder untere Körperhälfte auf einer Aufnahme abgebildet wurde. Diese Aufnahme wird von der deutschen als auch allen internationalen Leitlinien bei Fragestellung nach Kindesmisshandlung als obsolet angesehen. Durch die ungünstigen Projektions- und Belichtungsparameter ist die Beurteilung für spezifische Verletzungen bei Kindesmisshandlung mit einer solchen Ganzkörperaufnahme nur inkomplett möglich (Abb. 3).

Das Anlegen eines Gonadenschutzes ist, wenn immer möglich, nach BÄK-Leitlinie indiziert. Bei männlichen Patienten sollten die Keimdrüsen stets durch Hodenkapseln vor ionisierender Strahlung geschützt werden. Die Ovarblende kann im Rahmen des Röntgen-Skelettscreenings bei der Beckenaufnahme nicht angelegt werden, da sonst das knöcherne Becken nicht ausreichend zu beurteilen ist.

Die statistische Analyse erfolgte in Zusammenarbeit mit dem Institut für Medizinische Statistik, Informatik und Dokumentation und wurde mittels Kruskal-Wallis- und Mann-Whitney-U-Test durchgeführt.

## Ergebnisse

Insgesamt beteiligten sich 48 Institute mit 142 Fällen an der Studie. 114 Fälle aus 42 Instituten erfüllten die Einschlusskriterien. Ausgeschlossen wurden 28 Fälle, bei denen ein fehlerhaftes bzw. unzulässiges Dateiformat (*n* = 3), eine postmortale Untersuchung oder nur CT/MRT-Untersuchungen (*n* = 2) vorlagen. Weiterhin wurden ein Fall mit lediglich einer Röntgen-Thorax-Aufnahme und 21 Fälle jenseits von 36 Lebensmonaten (LM) ausgeschlossen.

Aus Universitätskliniken wurden 42 Fälle (37 %), aus Einrichtungen der Maximalversorgung ebenfalls 42 Fälle (37 %) und aus Kliniken der Regelversorgung 30 Fälle (26 %) ausgewertet. Elf kinderradiologische Fachabteilungen reichten 23 der 114 Fälle ein (20 %).

Das Alter der 114 betroffenen Kinder erstreckte sich von 0,75 Lebensmonaten (LM) bis 36 LM (Mittelwert: 9,7 LM; Median: 6 LM); 40 % (*n* = 46) der Patienten waren weiblich, 60 % (*n* = 68) männlich. Die Altersspanne der Jungen lag zwischen 0,75 und 33 LM und die der Mädchen von 1 bis 36 LM.

**Figure 4:**
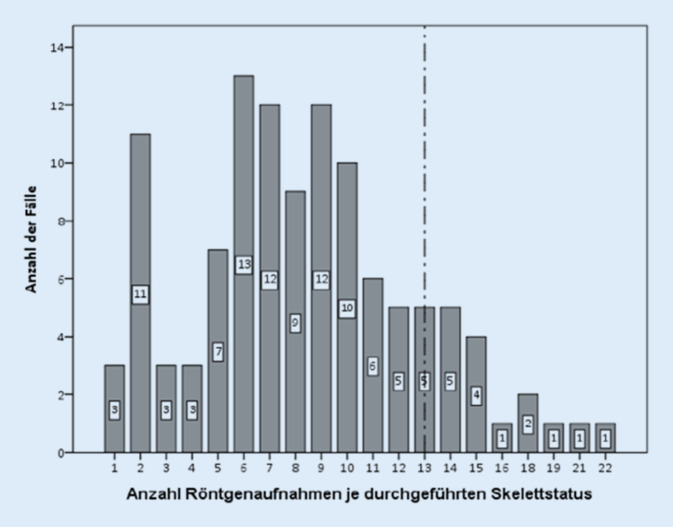

Insgesamt 958 Röntgenaufnahmen konnten in die Auswertung einbezogen werden. Minimal wurden eine, maximal wurden 22 Röntgenaufnahmen je Status angefertigt (Abb. 4). Im Mittel wurden 8,4 Aufnahmen je Verdachtsfall veranlasst.

In nur 10 % der Fälle (*n* = 12) wurde die von der damals geltenden S1-Leitlinie geforderte Anzahl an Röntgenaufnahme je Status veranlasst. Bei 10,5 % (*n* = 13) lag lediglich ein Babygramm vor (davon eine Komplettabbildung des kindlichen Körpers sowie 9 Aufnahmen der oberen, 3 der unteren Körperhälfte). Je nach Art der Institution sowie in Abhängigkeit einer Kinderradiologie variierte die Anzahl der veranlassten Aufnahmen je Skelettstatus. Zwar wurden in Universitätskliniken im Mittel (*n* = 9,1) mehr Röntgenaufnahmen je Status im Vergleich zu Maximal- (*n* = 7,9) oder Regelversorgern (*n* = 8,1) durchgeführt, jedoch zeigte sich hier kein signifikanter Unterschied (*p* = 0,3). Kliniken mit kinderradiologischer Fachabteilung fertigten signifikant mehr Röntgenaufnahmen je Status an (Mittelwert: 10,5 vs. 7,9 Aufnahmen; *p* = 0,01) als Einrichtungen ohne Kinderradiologie.

In 45,6 % (*n* = 52) der Fälle wurde der Gonadenschutz bei der jeweiligen Röntgenaufnahme (v. a. bei Aufnahmen des Beckens und der unteren Extremität) angelegt, in 42,1 % (*n* = 48) fehlte ein entsprechender Strahlenschutz. Eine differenzierte Darstellung aller Fälle zeigt Tab. 1, aufgeschlüsselt nach Geschlecht.

**Table 1:** 

Gonadenschutz	Alle Fälle		Männlich		Weiblich	
	*n*	%	*n*	%	*n*	%
Ja	52	45,6	36	52,9	16	34,8
Nein	48	42,1	26	38,3	22	47,8
Keine Röntgenaufnahme der jeweiligen Regionen vorliegend	14	12,3	6	8,8	8	17,4
*Gesamt*	*114*	*100,0*	*68*	*100,0*	*46*	*100,0*

**Figure 5:**
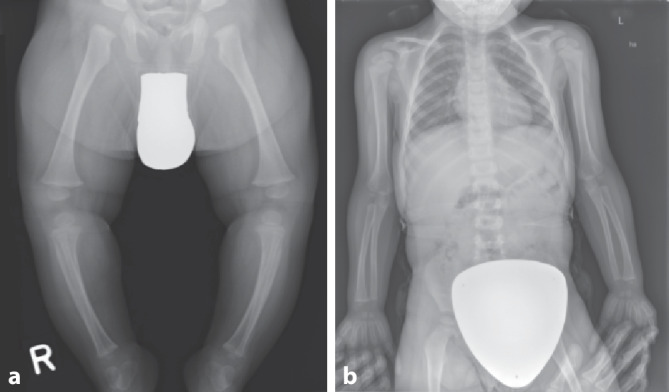

Ein Schutz durch eine Hodenkapsel bei Jungen fand sich in 52,9 % der Fälle (*n* = 36). Bei 38,3 % der Jungen (*n* = 26) und 47,8 % der Mädchen (*n* = 22) wurde auf den Gonadenschutz verzichtet wurde. Es muss hinzugefügt werden, dass der Gonadenschutz bei weiblichen Patienten die knöcherne Struktur des Beckens nicht überlagern darf. In Bezug auf die Überlagerung durch den Gonadenschutz werden die folgenden Abbildungen vergleichend angeführt (Abb. 5). In Abb. 5a sieht man den korrekt angelegten, adäquaten Gonadenschutz, der seine Aufgabe erfüllt und zu keiner Überlagerung relevanter Knochenstrukturen führt. Im Gegensatz dazu wurde in Abb. 5b zwar die Anlage eines entsprechenden Keimdrüsenschutzes realisiert, durch die unangebrachte Größe wurde jedoch das knöcherne Becken weitestgehend überlagert und die Aussagekraft des Skelettstatus somit erheblich eingeschränkt.

In der Auswertung der qualitativen Umsetzung zeigten sich institutionsabhängig Unterschiede im Score: Serien aus Universitätskliniken erreichen bei *p* < 0,001 einen signifikant höheren Scorewert (im Mittel 8,5 Punkte) als Status aus Maximal- (im Mittel 7,5 Punkte) oder Regelversorgerkliniken (im Mittel 8,0 Punkte). Zwischen Maximal- und Regelversorgern gab es keinen signifikanten Unterschied (*p* = 0,468). Ebenfalls kein signifikanter Unterschied in der Umsetzung konnte in Abhängigkeit einer kinderradiologischen Fachabteilung verzeichnet werden (beide Gruppen im Mittel 7,8 Punkte).

Bei 63 % (*n* = 72) der 114 eingeschlossenen Fälle erfolgte nach Angaben der beteiligten Institute laut Begleitfragebogen eine leitliniengerechte, bilddiagnostische Untersuchung. Bei 10 Fällen (9 %) wurde dies verneint. In 13 Einrichtungen (11 %) war die damals geltende S1-Leitlinie nicht bekannt. Bei 19 Fällen (16,4 %) wurden zum Kenntnisstand der Leitlinie keine Angaben gemacht. Der Vergleich der Angaben im Fragebogen mit der praktischen Umsetzung zeigte, dass lediglich in 11 der 72 Fälle (15 %) eine leitliniengerechte Bildfolge realisiert wurde. In den restlichen Fällen (*n* = 70, 85 %) konnte keine leitliniengerechte Darstellung nachgewiesen werden.

In den Begleitfragebögen wurde bei 62 % (*n* = 71) der Fälle angegeben, dass ein kompletter Skelettstatus durchgeführt wurde. In 21 % (*n* = 24) wurde diese Frage verneint. Angaben bezüglich dieser Frage fehlten bei 17 % (*n* = 17 %). Die Analyse der Fälle zeigte jedoch, dass von den 71 Fällen, in denen angegeben wurde, dass die Untersuchung komplett durchgeführt wurde, nur bei 5 (7 %) tatsächlich ein kompletter (d. h. leitliniengerechter) Skelettstatus vorlag.

Wurde bereits im Fragebogen ein inkompletter Status angegeben, konnte eine Begründung seitens des Institutes zusätzlich vermerkt werden. In 16 der 24 Fälle wurden Gründe für das Abweichen von der GPR-Leitlinie angegeben. Neben „Kind zu jung“ oder auf Grund der „zu hohen Strahlenbelastung“ wurden auch Gründe wie „Becken‑, Wirbelsäulen- sowie Hand- und Fußaufnahmen nicht Standard“ als auch „Röntgen nach Klinik“ als Begründung für den inkomplett durchgeführten Status angeführt.

## Diskussion

Die bildgebende Diagnostik nimmt in der Primärevaluation von nichtakzidentiellen Verletzungsfolgen im Kindesalter eine Schlüsselrolle ein und muss entsprechend qualitativ hochwertig durchgeführt und beurteilt werden [18–20]. Um für diese Fragestellung spezifische Verletzungsmuster und subtile Frakturzeichen zu erkennen, ist eine umfassende kinderradiologische Expertise notwendig [10], welche vielerorts fehlt [15]. Anders als in den USA wiesen lediglich 11 der 42 an dieser Analyse beteiligten Institute (26 %) eine explizit kinderradiologische Kompetenz auf [12]. In der aktuell gültigen S3+-Kinderschutzleitlinie empfiehlt die Gesellschaft für Pädiatrische Radiologie als Mitherausgeber grundsätzlich eine Doppelbefundung des Skelettscreenings durch zwei Kinderradiologen oder in der Diagnostik der Kindesmisshandlung erfahrene Radiologen [17].

Ziel dieser bundesweit angelegten Studie war es, eine Art Status-quo-Analyse zur röntgenologischen Diagnostik bei V. a. Kindesmisshandlung zu erhalten, wobei als Standard der Skelettstatus der damals gültigen S1-Leitlinie (GPR) genutzt wurde. Mit 142 Fällen, von denen 114 in die Analyse eingeschlossen werden konnten, lag die Rücklaufquote der aktuellen Studie mit 5,6 % deutlich unter der in international vergleichbaren Studien zu verzeichnenden Beteiligung. Kleinman et al. (2004), die gezielt spezialisierte Zentren in den USA untersuchten, erreichten eine Rate von 88 % [12], van Rijn et al. (2009) immerhin 38 % aller informierten Mitglieder der Niederländischen Gesellschaft für Radiologie [15]. Eine britische Studie, in der sowohl Einrichtungen mit radiologischen als auch pädiatrischen Abteilungen informiert wurden und die damit am ehesten dem vorliegenden Studiendesign entsprach, zeichnete sich durch eine Beteiligung von 35 % aus [10].

Ähnlich wie bei bislang publizierten internationalen Studien zeigte das Untersuchungsmaterial eine große Spannweite in der Anzahl angefertigter Röntgenaufnahmen mit deutlichen Abweichungen von den mindestens 13 Einzelaufnahmen, die in der damals geltenden deutschen S1-Leitlinie gefordert wurden. Mit durchschnittlich 8,4 Röntgenbildern wurden weniger Aufnahmen veranlasst als in Vergleichsstudien [10–12, 14, 15]. In der von Offiah und Hall initiierten Studie in Großbritannien wurden initial 10 Aufnahmen je Status (Spannweite: 2–13 Aufnahmen) dokumentiert [11]. Nach Publikation einer Leitlinie, die ein Mindestmaß von 20 Aufnahmen forderte, nahm die Anzahl auf durchschnittlich 16,5 Aufnahmen zu. [14].

Van Rijn et al. (2009) konnten zeigen, dass die Anzahl von Röntgenaufnahmen von der Größe der Institution abhängig ist. Er konnte mit durchschnittlich 16,2 Aufnahmen je Status in der universitären Versorgung signifikant höhere Werte verzeichnen als bei Röntgenuntersuchungen in nichtuniversitären Einrichtungen (8,2 Aufnahmen; [15]). Die aktuelle Studie zeigt bei vorhandener kinderradiologischer Fachabteilung signifikant höhere Werte in der Anzahl angefertigter Röntgenaufnahmen.

Unterschiede in der qualitativen Durchführung des Skelettstatus zwischen verschiedenen Instituten wurden anhand der ermittelten Scorewerte deutlich. Während die mittleren Werte im universitären Bereich signifikant höhere Punktzahlen im Vergleich zu Regel- und Maximalversorgung erzielten, konnte zwischen Instituten mit bzw. ohne kinderradiologische Fachabteilung in den ermittelten Punktwerten kein weiterer, signifikanter Unterschied aufgezeigt werden. Das kann durch Differenzen in der Gruppengröße begründet werden. Während ohne kinderradiologische Fachabteilung 91 Fälle vorlagen, bestand die Fallgruppe mit kinderradiologischer Fachabteilung aus lediglich 23 Untersuchungen. Zudem beinhalteten beide Gruppen Fälle von Regel- und Maximalversorgern sowie aus Unikliniken. Es kann deswegen davon ausgegangen werden, dass Schwankungen der Punktzahl in der Gruppe ohne kinderradiologische Fachabteilung besser ausgeglichen werden konnten.

Vor dem Hintergrund der diffizilen Fragestellung und damit verbunden häufig nur subtilen skeletalen Veränderungen haben van Rijn und Sieswerda-Hoogendoorn [20] die Forderung formuliert, die Befundung des Skelettstatus entsprechend geschulten Fachkräften zu überlassen bzw. diese aktiv in die Befundung zu involvieren. Neben hoher Bildqualität ist in diesem Fall von einer qualitativ besseren Umsetzung der Skelettstatus und höherer Leitlinientreue auszugehen [20]. Weiterhin lässt sich aus den vorliegenden Ergebnissen die Empfehlung zur Zweitbefundung ableiten. Ähnlich der Mammadiagnostik, bei der für die Mammographie eine Doppel-Facharzt-Befundung vorgeschrieben ist, sollte zukünftig bei Verdacht auf Kindesmisshandlung der röntgenologische Skelettstatus durch einen weiteren, speziell ausgebildeten Kinderradiologen bzw. durch einen Radiologen mit Schwerpunkt pädiatrische, muskuloskeletale Bildgebung begutachtet werden, bevor die abschließende radiologische Diagnose gestellt wird. Die bildgebende Diagnostik ist im Fall der Misshandlung die Basis der gutachterlichen Einschätzung der (Kinder‑)Radiologie und Rechtsmedizin und hat erhebliche Bedeutung aus forensischer Sicht. In der Mehrzahl der für die Studie eingereichten Fälle erfolgte eine unvollständige Darstellung des Skeletts. Angegebene Begründungen hierfür implizieren, dass das Bewusstsein für spezifische Frakturtypen einer nichtakzidentellen Verletzung stärker geweckt werden muss [21]. Angaben wie „Röntgen nach Klinik“ sind bzgl. der Fragestellung Kindesmisshandlung inakzeptabel, da mittels Skelettscreening okkulte bzw. ältere Frakturen detektiert werden sollen, die „klinisch stumm“ verlaufen sind.

Unsere Studie verdeutlicht, dass es zum damaligen Zeitpunkt (2009–2011) in Deutschland kein allgemein bekanntes, standardisiertes Vorgehen bei Verdacht auf Kindesmisshandlung gab. Die Untersuchung von Offiah und Hall aus dem Jahr 2003 vor Revision der britischen Leitlinie zeigte eine vergleichbare Inhomogenität und Mängel in der Umsetzung des geforderten Skelettstatus [11]. Den positiven Einfluss einer entsprechenden Bekanntmachung konnte die britische Folgestudie aus dem Jahr 2008 von Swinson et al. nach Veröffentlichung der neuen Leitlinie zeigen [12]. In diesem Kontext bleibt zu prüfen, ob die mittlerweile in Deutschland publizierte S3+ Kinderschutzleitlinie zu einem einheitlichen Vorgehen in der bildgebenden Diagnostik bei Verdacht auf Kindesmisshandlung führen wird. Die hier vorliegende Studie auf Basis der S1-Leitlinie der GPR kann dabei als Ausgangspunkt einer neuen deutschlandweiten Studie zur Leitlinientreue dienen, um hoffentlich positive Entwicklungen in der Leitlinienumsetzung aufzuzeigen.

### Limitationen

Durch die geringe Rücklaufquote konnte nur ein gewisser Anteil der im Untersuchungszeitraum durchgeführten Röntgenuntersuchungen in Deutschland analysiert werden. Das Ergebnis der Studie unterlag zudem einem Selektionsbias, welcher durch die auf freiwilliger Basis beruhende Beteiligung der Institute bedingt ist. Verstärkt wurde die Verzerrung dadurch, dass wahrscheinlich von den einsendenden Instituten eine Vorauswahl an Fällen, welche in die Studie einfließen sollten, getroffen wurde.

### Ausblick

Um eine hochwertige radiologische Diagnostik im Rahmen der Evaluation von Säuglingen und Kindern bei Misshandlungsverdacht gewährleisten zu können, wird in Leitlinien die Durchführung eines Skelettscreenings empfohlen. Für Deutschland ist ein an aktuellen Leitlinien orientiertes diagnostisches Vorgehen zu fordern. In dieser Studie aufgezeigte Abweichungen von der Leitlinie sind künftig zu minimieren. Der Kenntnisstand der verantwortlichen Radiologen um Aspekte dieser speziellen Fragestellung kann durch eine bessere Publikation und Verbreitung der S3+ Kinderschutzleitlinie verbessert werden.

Bei geringer Anzahl kinderradiologischer Spezialisten ist die Etablierung von Referenzzentren zu diskutieren. Durch Zweitbefundung und Beratung in der Durchführung können diagnostische Aussagekraft und Qualität der Röntgenuntersuchungen verbessert und Kollegen, die selten mit dieser Indikationsstellung konfrontiert sind, unterstützt werden.

Eine Neuauflage unserer Studie zur erneuten Evaluation der Leitlinientreue bei V. a. Kindesmisshandlung ist nach Einführung der S3+-Kinderschutzleitlinie unbedingt wünschenswert. Unter Nutzung aller Gremien der radiologischen, rechtsmedizinischen und pädiatrischen Fachgesellschaften und entsprechender Medienarbeit muss das Bewusstsein für diese spezielle Fragestellung geschärft werden. Unter Einbeziehung von Kollegen aus Österreich und der Schweiz kann die Teilnahmezahl erhöht und ein Abbild des radiologischen Vorgehens im deutschsprachigen Raum bei V. a. Kindesmisshandlung gegeben werden.

## Fazit für die Praxis

- Bestandteile des Skelettstatus nach S1-Leitlinie und Aufnahmeeinstellungen waren dem verantwortlichen Personal nur unzureichend bekannt.
- Der durchgeführte röntgenologische Skelettstatus in Deutschland zeichnet sich in quantitativer und qualitativer Umsetzung durch hohe Variabilität aus.
- Kinderradiologische Fachabteilungen fertigen signifikant mehr Röntgenaufnahmen je Skelettstatus an (*p* < 0,05); in der Umsetzung zeigen Abteilungen in den Universitätskliniken die höchste Qualität.
- Eine Etablierung kinderradiologischer Referenzzentren für Zweitbefundung und Beratung zum Vorgehen nach S3+ Kinderschutzleitlinie zur Qualitätsverbesserung und -sicherung ist erforderlich.
- Eine erneute Studie zur Evaluation der Leitlinientreue nach Publikation der neuen S3-Kinderschutzleitlinie 2019 wäre wünschenswert.
